# Far-reaching effects of tyrosine64 phosphorylation on Ras revealed with BeF_3_^–^ complexes

**DOI:** 10.1038/s42004-024-01105-6

**Published:** 2024-01-31

**Authors:** Patrick Baumann, Yi Jin

**Affiliations:** 1 School of Chemistry, Cardiff University, Park Place, Cardiff, CF10 3AT UK; 2 Department of Chemistry, School of Natural Sciences, Faculty of Science and Engineering, University of Manchester, M13 9PL, Manchester, UK; 3 Present Address: Manchester Institute of Biotechnology, University of Manchester, 131 Princess Street, Manchester, M1 7DN UK

**Keywords:** Enzyme mechanisms, X-ray crystallography, Mechanism of action, Solution-state NMR

## Abstract

Tyrosine phosphorylation on Ras by Src kinase is known to uncouple Ras from upstream regulation and downstream communication. However, the mechanisms by which phosphorylation modulates these interactions have not been detailed. Here, the major mono-phosphorylation level on tyrosine64 is quantified by ^31^P NMR and mutagenesis. Crystal structures of unphosphorylated and tyrosine64-phosphorylated Ras in complex with a BeF_3_^−^ ground state analogue reveal “closed” Ras conformations very different from those of the “open” conformations previously observed for non-hydrolysable GTP analogue structures of Ras. They deliver new mechanistic and conformational insights into intrinsic GTP hydrolysis. Phosphorylation of tyrosine64 delivers conformational changes distant from the active site, showing why phosphorylated Ras has reduced affinity to its downstream effector Raf. ^19^F NMR provides evidence for changes in the intrinsic GTPase and nucleotide exchange rate and identifies the concurrent presence of a major “closed” conformation alongside a minor yet functionally important “open” conformation at the ground state of Ras. This study expands the application of metal fluoride complexes in revealing major and minor conformational changes of dynamic and modified Ras proteins.

## Abstract (web summary)

Protein tyrosine kinase Src is known to phosphorylate Ras protein on tyrosine32 and 64, and uncouple Ras from the signaling cascade, however, the mechanisms through which phosphorylation modulates these interactions remain poorly understood. Here, the authors quantify the major mono-phosphorylation level on tyrosine64 by ^31^P NMR and mutagenesis, and reveal the key conformational changes of phosphorylated Ras using BeF_3_^−^ complexes by X-ray crystallography and ^19^F NMR, providing new mechanistic and conformational insights into Ras dynamics regulation.

## Introduction

The three human oncogenes, HRas, NRas and KRas, encode highly related membrane-bound Ras protein isoforms that act as “molecular switches” cycling between GDP-bound and GTP-bound forms^1,2^. Guanine nucleotide-exchange factors (GEFs) are recruited to promote the intrinsically slow GDP to GTP nucleotide exchange, which is coupled to distinct conformations at Switch I (residues 30–38) and Switch II (residues 59–72) near GDP/GTP binding site^3^. The GTP-bound active form has a high affinity to downstream effector proteins, such as Raf, and triggers the mitogen-activated protein kinase (MAPK) pathway^3^. This GTPase signalling is terminated by accelerated GTP hydrolysis catalysed by GTPase-activating proteins (GAPs)^4^. Deregulation of the GTPase cycle in Ras is commonly associated with cancer initiation and progression^5^.

Protein tyrosine kinase Src was recently shown to phosphorylate Ras proteins on tyrosine residues 32 and 64^6,7^. It was first reported that Src predominantly phosphorylates Y32 in the GDP-bound GST-tagged Ras and was thought to allow the more favoured displacement of downstream effector Raf by GAP, which then increases GAP-catalysed GTP hydrolysis^6,8^. That led to a focused investigation^8^ on the effect of SHP2-mediated dephosphorylation, only on Y32. However, it was later found that Ras in both GDP and GTP-bound forms can be phosphorylated on Y64 in Switch II faster than Y32 in Switch I by Src which reduces the activity of GAP^7^. Therefore, phosphorylation of these sites has been proposed as the mechanism for uncoupling phosphorylated Ras from upstream regulation in vivo, especially by attenuation both of the nucleotide exchange catalysed by GEF and of GTP hydrolysis activity catalysed by GAP^7^. Particularly, Y64 has been identified as one of the major hotspots for effector interaction^9^. Nonetheless, contradictory findings regarding Src-mediated phosphorylation levels on Y32 and Y64 have given rise to divergent hypotheses. These hypotheses revolve around whether phosphorylation enhances or diminishes GAP binding, and subsequently, how this modulation influences GTP hydrolysis rate and downstream effector interactions^6,7^. To date, only a handful of computational investigations have centred on the conformational ramifications of mono-phosphorylation on Y32 or dual phosphorylation on both Y32 and Y64 concerning effector and inhibitor interactions^10–12^. Nonetheless, the accurate quantification of each tyrosine phosphorylation level and the specific ways in which phosphorylation influences intrinsic Ras nucleotide exchange, GTP hydrolysis, and interactions with downstream effectors all remain elusive^7,13^.

Pre-hydrolysis state structures of Ras provide conformational information for substrate binding and explain the molecular origin of intrinsic GTP hydrolysis and nucleotide exchange. They have been depicted by numerous structures of Ras co-crystallised with non-hydrolysable GTP analogues, including guanosine 5’-[β,γ-imido] triphosphate (GMPPNP), guanosine 5’-[β,γ-methylene] triphosphate (GMPPCP), and guanosine 5’-*O*-[γ-thio] triphosphate (GTPγS). However, because of their non-isopolar chemical changes^14^, these compounds have modified electron densities on their γ-phosphate oxygens resulting in changed protonation states in bound complexes for Ras and significantly modified H-bonding compared to the true ground state of GTP in Ras^15,16^. Metal fluoride complexes (MF_x_) have been used extensively in structural biology to monitor conformational changes and address the activation origin of proteins^17^. The ground state analogue (GSA) BeF_3_^−^ (Protein Data Bank (PDB) Chemical ID BEF) mimics the tetrahedral geometry of phosphate before or after phosphoryl transfer. ^19^F NMR as a highly sensitive technique offers a more direct spectroscopic approach to provide a detailed picture of the charge distribution in the ground and transition states for P–O bond through MF_x_ complexes mimicking the γ-phosphate of GTP in GTPases^18,19^, γ-phosphate of ATP in kinases^20,21^, and bacterial phosphatases and phosphomutases^22–27^. ^19^F NMR has also successfully reported the conformational changes that regulate the phosphatase activity of bacterial histidine kinase with BeF_3_^−^ complexes^26,28^. For Ras, GDP-BeF_3_^–^ could provide a valuable alternative ground state conformation because all three fluorine atoms are capable of accepting H-bonds as a fully deprotonated γ-phosphate in GTP^15^. However, no x-ray structures of the BeF_3_^–^ GSA complex for Ras have been deposited in the PDB to date.

We here show by mutagenesis and ^31^P NMR the mono-phosphorylation level on Y64 in Ras is significantly higher than for other mono- and double-phosphorylated species, ruling out Src being a dual-kinase for Ras. Our work delivers pioneering BeF_3_^–^ GSA complex x-ray structures for unphosphorylated (Ras_WT_) and Y64-monophosphorylated Ras (Ras_pY64_) that unveil novel “closed” ground state conformations for the intrinsic hydrolysis of Ras, which are distinct from other non-hydrolysable GTP analogue-bound structures. The phosphorylation on Y64 in Switch II triggers a cascade of conformational changes beyond the active site, potentially impairing the binding of Ras to downstream effector Raf and offering an alternative mechanism to previous proposals^12^. Combined with high-resolution x-ray crystal structures, the subtle yet significant ^19^F NMR chemical shifts and linewidth changes between Ras_WT_-GDP-BeF_3_^–^ and Ras_pY64_-GDP-BeF_3_^–^ GSA complexes offer viable insights into decreased intrinsic GTPase rate and increased nucleotide exchange rate on phosphorylation^6^. Our study expands the evidence-based understanding of post-translational modification (PTM)-induced conformational changes of Ras. Furthermore, it underscores the capability of ^19^F NMR to detect unnoticed conformations and conformational alterations in high-resolution structures, demonstrated here *via* MF_x_ complexes.

## Results and discussion

### Phosphorylation of Y64 is the main Ras site for Src kinase

**Fig. 1:**
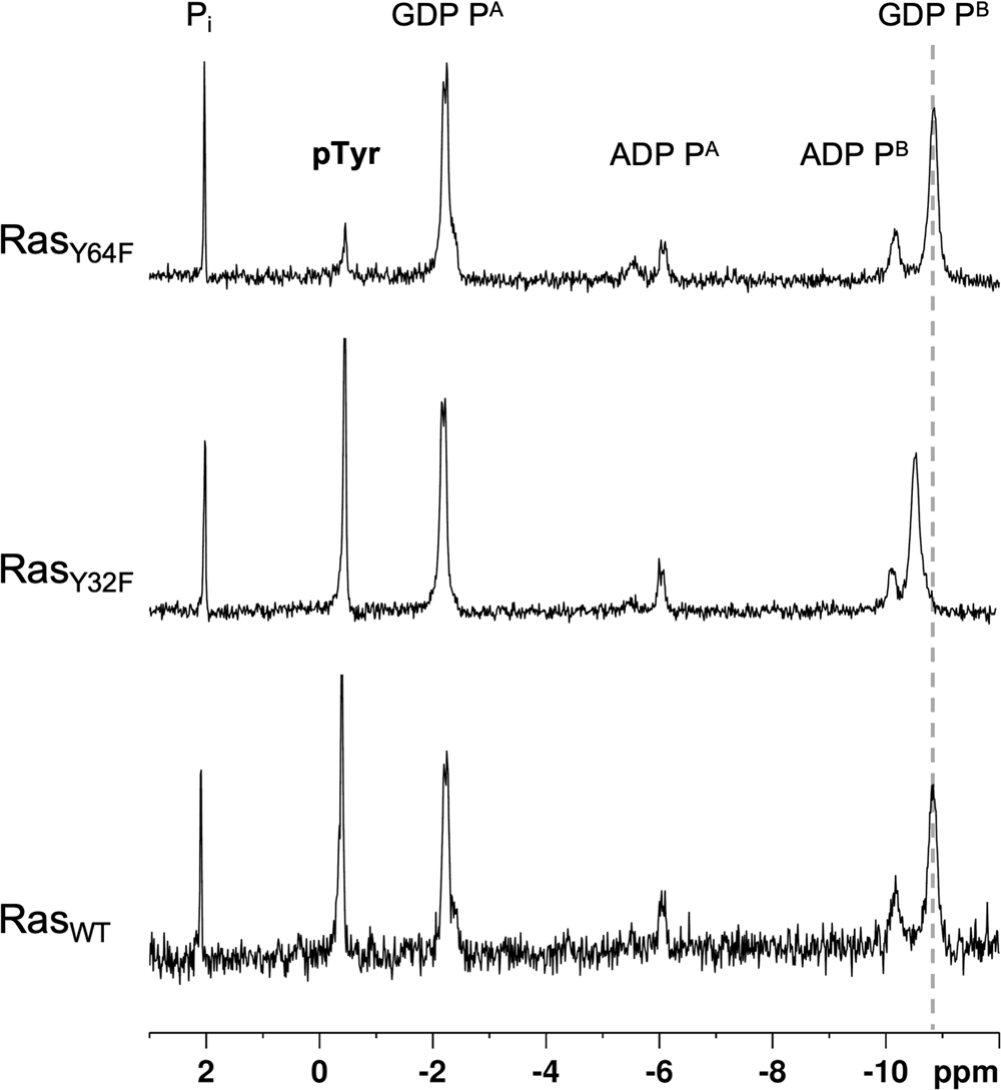
^31^P NMR spectra of Src-mediated Ras phosphorylation. Ras_WT_, Ras_Y32F_ or Ras_Y64F_ (1.0 mM) were incubated with Src (20 µM) and phosphorylation buffer (ATP 5 mM, Tris-HCl 25 mM, pH = 7.6, NaCl 200 mM, MgCl_2_ 5 mM) at 4 °C for 16 h. ATP, ADP and inorganic phosphate were largely removed by buffer exchange to avoid signal overlap for clarity.

Expression tags have been shown to affect the site preference of tyrosine phosphorylation of Ras^6^. Thus, we recombinantly generated un-tagged Ras. A phosphorylation assay of the GDP-bound HRas (hereafter Ras) by Src catalytic domain was set up by using physiologically relevant concentrations of Mg^2+^ and ATP^29^. Mass spectroscopy (MS) analysis detected unphosphorylated, mono-, and double-phosphorylated species (Supplementary Figure 1). To quantify the site-specific phosphorylation, ion exchange chromatography was used to separate major mono-phosphorylated Ras species from non- and double-phosphorylated ones; double-phosphorylated species only show minor peaks (Supplementary Figure 2). The sole mono-phosphorylated Ras fraction was trypsin digested and MS detected phosphorylated peptide fragments only on Y64 (Supplementary Figure 3). To specifically quantify phosphorylation levels, Y32F and Y64F variants of Ras, Ras_Y32F_ and Ras_Y64F_, were also prepared and phosphorylated by Src under the same condition as for Ras_WT_. ^31^P NMR shows the integration of the resonance from the phosphorylated tyrosine (–0.4 ppm) is reduced by 85% for the Ras_Y64F_ variant compared to Ras_WT_ and Ras_Y32F_ variant (Fig. 1). This reveals that Y64 is the main phosphorylation target of Src. It conflicts with the conclusion in previous work that Y32 is the major phosphorylation site, possibly due to the in vitro phosphorylation assay was there carried out with GST-tagged HRas^6^. In addition, the resonance of P^B^ ^30^ of the bound GDP in the Y64-phosphorylated Ras_Y32F_ variant moved downfield by 0.5 ppm relative to the less-phosphorylated Ras_Y64F_ variant, indicating the mutation of Y32 to F has induced local conformational changes around the P-loop (Fig. 1)^31^.

### A GDP-BeF_3_^–^ complex delivers conformational changes different from non-hydrolysable GTP analogues

**Fig. 2:**
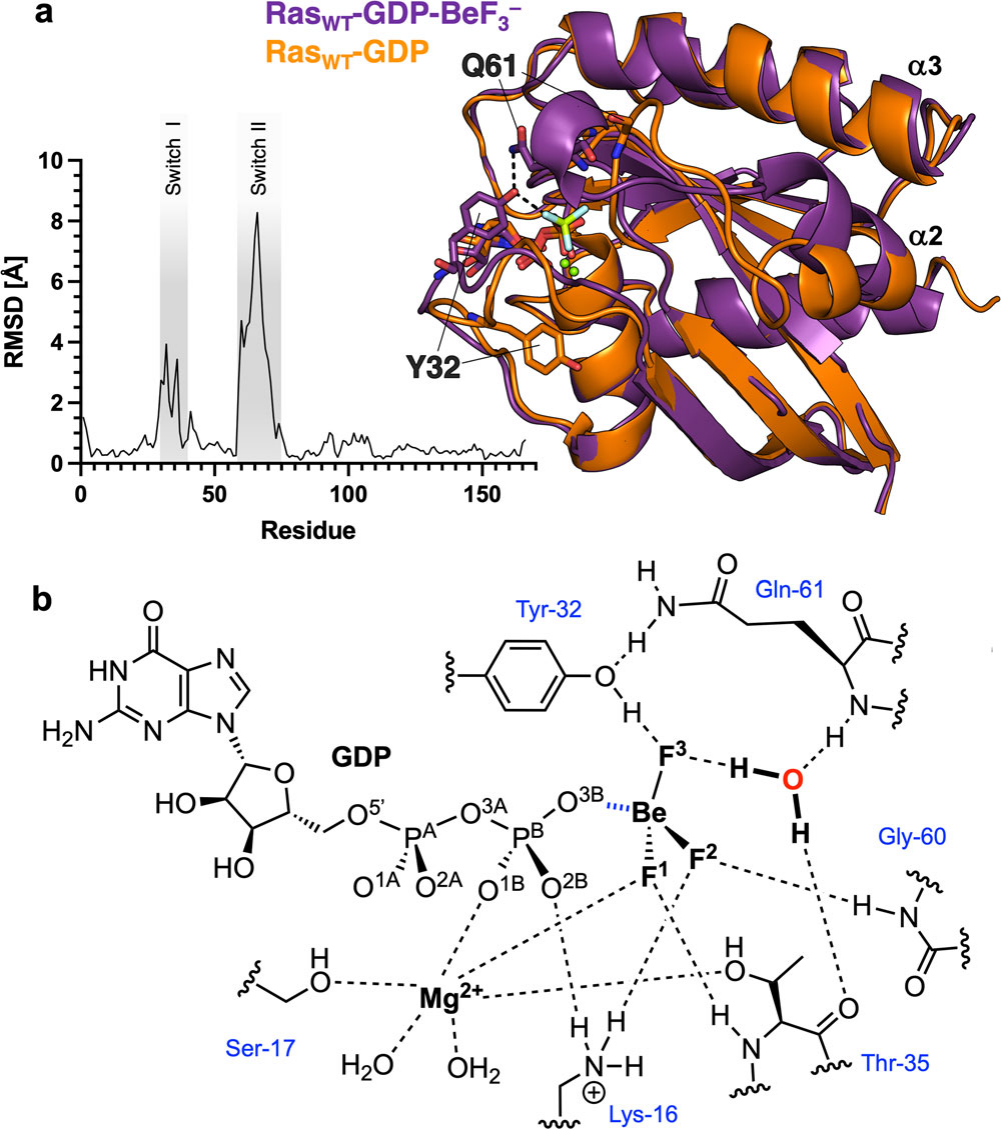
Ras_WT_-GDP-BeF_3_^–^ GSA complex. **a** Overlay of Ras_WT_-GDP (orange, PDB: 4Q21) and Ras_WT_-GDP-BeF_3_^–^ (purple, PDB: 8CNJ). RMSD for the structure alignment is plotted for each residue. The major conformational changes are induced in the Switch I and Switch II regions. **b** H-bond interactions for the Ras-GDP-BeF_3_^–^ GSA complex were observed in its structure.

**Fig. 3:**
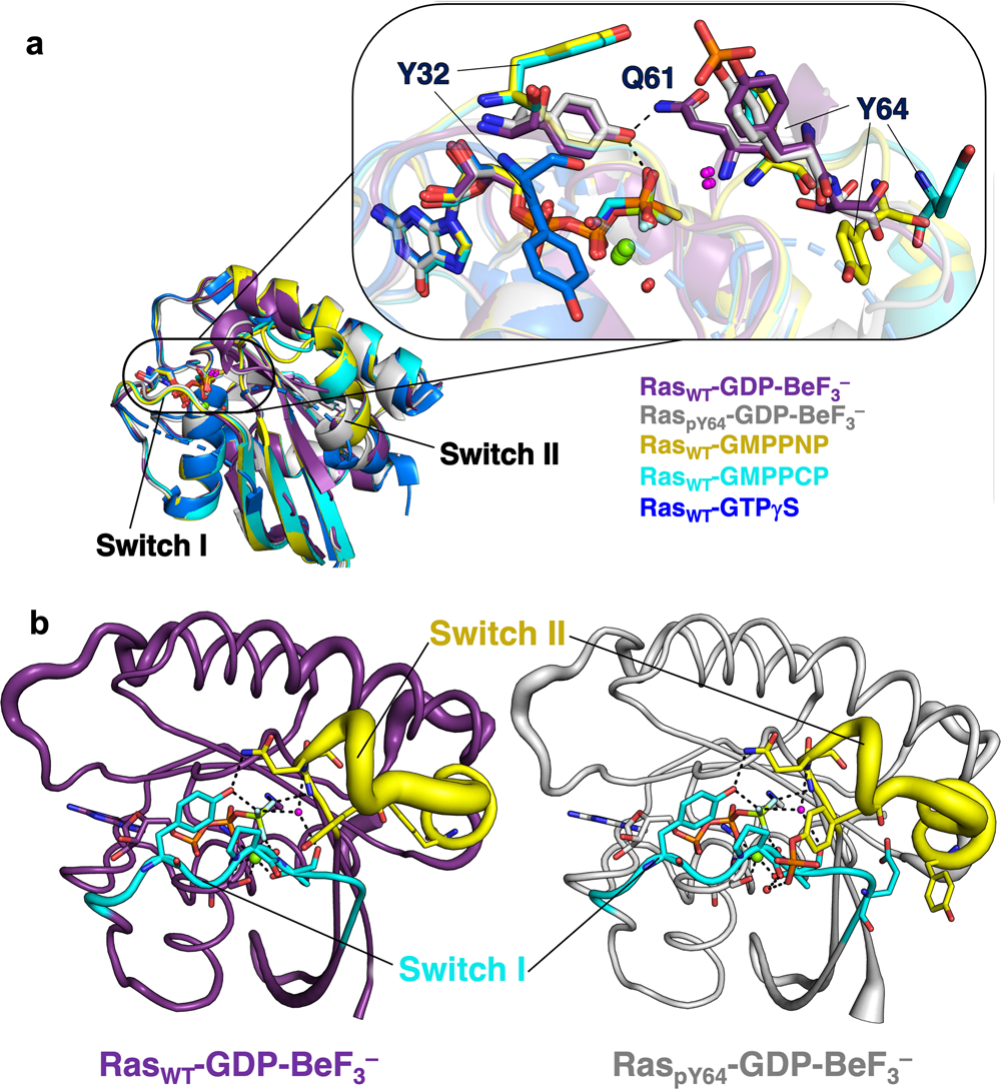
Comparison of GDP-BeF_3_^–^ GSA complex structures of Ras to other Ras GSA structures with non-hydrolysable GTP analogues or caged GTP analogues. **a** Overlay of different Ras structures (Ras_WT_-GDP-BeF_3_^–^ PDB: 8CNJ, Ras_pY64_-GDP-BeF_3_^–^ PDB: 8CNN, Ras_WT_-GTP PDB: 1QRA, Ras_WT_-GMPPCP PDB: 121P, Ras_WT_-GMPPNP PDB: 5P21, Ras_WT_-GTPγS PDB: 5VQ6) show direct coordination of Y32 for the Ras-BeF_3_^–^ GSA structures and a more “open” Y32 conformation for the Ras structures complexed with non-hydrolysable GTP analogues. **b** B-factor putty models of Ras_WT_-GDP-BeF_3_^–^ (purple) and Ras_pY64_-GDP-BeF_3_^–^ (grey) show increased mobility for the Switch II region.

After unsuccessful attempts of crystallising a BeF_3_^–^ GSA complex by co-crystallising Ras_WT_-GDP with Be^2+^ and F^–^, we adopted a new approach by soaking Ras_WT_-GDP apo crystals with 50 mM BeCl_2_ and 0.8 M NH_4_F for 1 to 2 min followed by flash freezing. We successfully obtained a BeF_3_^–^ structure for Ras_WT_-GDP-BeF_3_^–^ GSA complex (1.35 Å resolution, PDB: 8CNJ, Table 1, Supplementary Data 1). In this complex, the α2-helix (residues 66–74), which overlaps with Switch II in sequence, deviates from the one in the Ras-GDP structure by 42° but it adopts a conformation highly similar to the GTP- or GTP analogue-bound unphosphorylated Ras structures with an angle difference of <7° (Fig. 2a, Supplementary Figure 4, Table 2). This demonstrates this Ras_WT_-GDP-BeF_3_^–^complex is mimicking a GTP-bound GSA state. This is also echoed by the high B-factor of Switch II observed, showing more mobility around the Switch II region than the rest of the protein (Fig. 3b), similar to the observation in the structure for the RhoA/RhoGAP product complex with both the GDP and P_i_ bound, where the inorganic phosphate was also introduced by ligand soaking^32^. However, different from the GMPPCP (PDB: 121P) or GMPPNP-bound (PDB: 5P21) structures of Ras_WT_, our BeF_3_^–^ structure has well-defined electron densities for both Switch I and Switch II, in which Y32 donates an H-bond to F^3^ and also accepts an H-bond from Q61 to the phenolic-OH of Y32. Thus, both Y32 and Q61 adopt a “closed” conformation (Fig. 2b). This is significantly different from the Y32 and Q61 conformations in other GSA structures of Ras with non-hydrolysable GTP analogues or by cryo techniques from a caged GTP analogue^33^ (Fig. 3a). In the Ras-GMPPCP (PDB: 121P) and Ras-GMPPNP (PDB: 5P21, 4RSG) GSA complexes, the Y32 and Q61 are in a completely “open” conformation. In the Ras-GTPγS GSA complex (PDB: 5VQ6), Y32 and Q61 are both disordered and incapable of providing any information.

**Table 1:** X-ray data collection, processing, and refinement statistics.

	HRas_pY64_-GDP apo	HRas_WT_-GDP-BeF_3_^–^	HRas_pY64_-GDP-BeF_3_^–^
PDB	8BWG	8CNJ	8CNN
**Crystal Data**			
Wavelength	0.976 Å	0.976 Å	0.976 Å
Space group	H 3 2	P 21 21 21	H 3 2
*a*, *b*, *c* (Å)	92.66, 92.66, 119.32	45.43, 51.96, 136.15	87.77 87.77 132.38
α, β, γ (°)	90.00, 90.00, 120.00	90.00, 90.00, 90.00	90.00, 90.00, 120.00
Resolution (Å)	47.92–1.32	48.59–1.35	49.92–1.48
*R*_merge_	0.041	0.056	0.033
*I* / sig(*I*)	28.6 (1.1)	13.7 (1.4)	15.8 (1.2)
CC(1/2)	1.000 (0.577)	0.999 (0.598)	1.000 (0.559)
Completeness (%)	99.2 (91.0)	96.2 (68.4)	100.0 (100.0)
**Refinement**			
Unique reflections	45988 (2073)	69077 (2387)	32936 (1622)
*R*_work_ / *R*_free_	0.135 / 0.174	0.144 / 0.183	0.138 / 0.176
No. atoms			
Protein	1335	2707	2669
Ligand/ion	33 / 1	64 / 2	140 / 1
Water	131	260	123
*B*-factors			
Protein	25.65	20.99	24.07
Ligand/ion	24.28 / 20.97	14.16 / 14.95	39.8 / 16.43
Water	37.45	34.49	36.09
RMS deviations			
Bond lengths (Å)	0.0097	0.0137	0.0118
Bond angles (°)	1.607	1.750	1.637

Values in parentheses are for the highest-resolution shell.

**Table 2:** Angle differences in α2-helix of the Switch II region for Ras structures.

	Ras_pY64_-GDP-BeF_3_^–^	Ras_WT_-GDP-BeF_3_^–^	Ras_WT_-GTP	Ras_WT_-GMPPNP	Ras_WT_-GMPPCP	Ras_pY64_-GDP
Ras_WT_-GDP	34.57°	41.99°	37.13°	43.73°	42.09°	2.67°
Ras_pY64_-GDP	32.41°	40.44°	35.35°	41.99°	43.80°	
Ras_WT_-GMPPCP	12.89°	3.29°	6.82°	0.65°		
Ras_WT_-GMPPNP	12.43°	3.80°	6.65°			
Ras_WT_-GTP	7.55°	6.27°				
Ras_WT_-GDP-BeF_3_^–^	13.53°					

Ras_pY64_-GDP-BeF_3_^–^, PDB: 8CNN; Ras_WT_-GDP-BeF_3_^–^, PDB: 8CNJ; Ras_WT_-GTP, PDB: 1QRA (caged-GTP analogue); Ras_WT_-GMPPNP, PDB: 121P; Ras_WT_-GMPPCP, PDB: 5P21; Ras_pY64_-GDP, PDB: 8BOS; Ras_WT_-GDP, PDB: 4Q21. Data are colours coded by the level of difference for clarity.

Since the overall conformations of the GTP analogues are close to that of our BeF_3_^–^ complex structure, this closure of Y32 and Q61 must relate to the electron density of the surrogate γ-phosphoryl group atoms. Numerous computational studies based on these GTP analogue structures have concluded that the slow, intrinsic hydrolysis of GTP by Ras involves a solvent-assisted pathway via a “2 W” mechanism^34,35^, with partial support from a GTP-bound structure from a caged GTP analogue^33^. Calculations of conformational effects by PTMs and mutations of Ras have also been based on structures using non-hydrolysable GTP analogues^36,37^. Therefore, it is highly significant that in our Ras_WT_-GDP-BeF_3_^–^ GSA complex structure there is no second water molecule within 7.8 Å of P^G^. Only the isolated nucleophilic water is in a near-attack conformation (NAC) at P^G^ along with the two waters closely coordinated to the octahedral catalytic Mg^2+^. This NAC water has an in-line angle (O^3B^–Be–Ow) 161° and is 3.5 Å from the beryllium atom. It donates an H-bond directly to T35 and F^3^ and accepts a H-bond from Q61 (Fig. 2b). This highlights the different conformational details provided by GDP-BeF_3_^–^ and GTP analogues might lead to different mechanistic conclusions when alternative H-bonding patterns from residues G60 and Q61 are included in the QM zone^15,38–40^.

### ^19^F NMR of BeF_3_^–^ GSA complexes capture both “open” and “closed” conformations

**Fig. 4:**
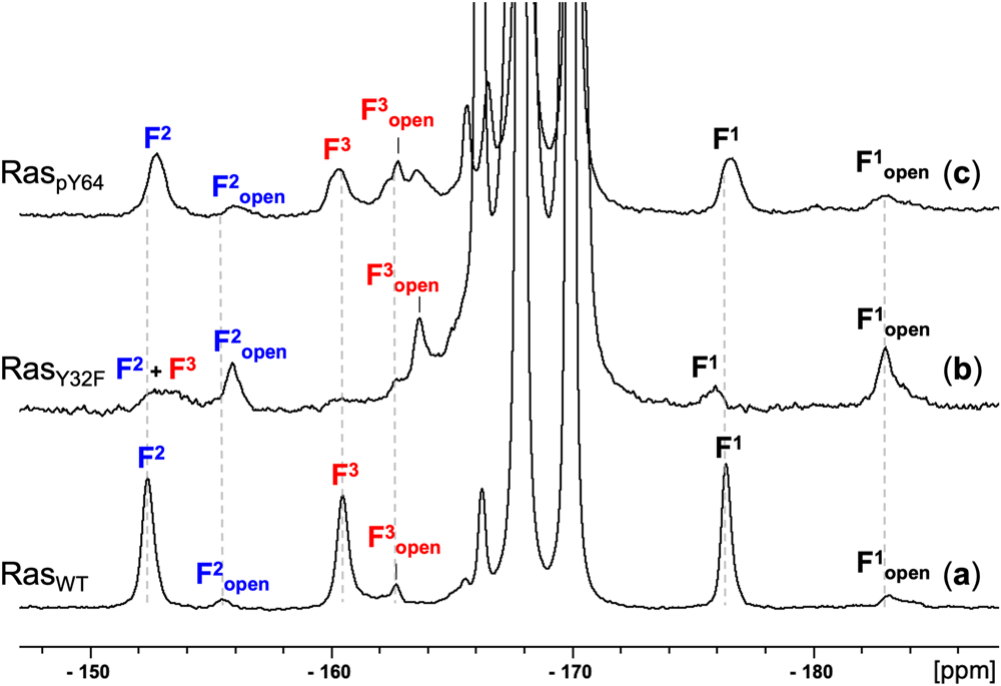
^19^F NMR spectra of GDP-BeF_3_^–^ GSA complexes. **a** Ras_WT_-GDP-BeF_3_^–^ complex, **b** Ras_Y32F_-GDP-BeF_3_^–^ complex, and **c** HRas_pY64_-GDP-BeF_3_^–^ complex. All spectra were acquired with presaturation on the unbound fluoride resonance at –120 ppm. Samples consisted of 0.8–1.0 mM Ras protein in buffers of 25 mM Tris-HCl pH = 7.0 with 10% D_2_O with 3 mM BeCl_2_, 30 mM NH_4_F, and 5 mM MgCl_2_.

Compared to crystal structures providing a snapshot of a conformation, solution ^19^F NMR has the unique advantage of detecting subtle conformational changes via chemical shift changes^17,41^. It can also capture minor conformations in phosphoryl transfer proteins that switch between several conformations because of its high sensitivity^25,27^. Furthermore, by comparing the ^19^F chemical shifts of the same resonance measured in 10% D_2_O and 90–100% D_2_O, solvent-induced isotope shift (SIIS) can be calculated which accurately reflects the number and orientation of H-bond donors around each fluorine^42^. To assess whether the structural changes reflected by the BeF_3_^–^ GSA structure are genuinely caused by Y64 phosphorylation and not by crystallographic artefacts, we investigated this BeF_3_^–^ complex in solution ^19^F NMR with presaturation on free fluoride resonance at −120 ppm. The pre-saturated ^19^F NMR spectrum of the Ras_WT_-GDP-BeF_3_^–^ complex shows three major well-resolved peaks for the protein-bound BeF_3_^–^ moiety (Fig. 4a), outside the known range (–163 to –170 ppm) for free BeF_*x*_ species. The signal of F^1^ at −176.3 ppm is significantly more upfield than the other resonances and has 0.3 ppm SIIS, indicating that it has a relatively high electron density and low proton density. It is thus assigned to F^1^, coordinated to the catalytic magnesium^25,26,28^. F^2^ at −152.3 ppm and F^3^ at −160.4 ppm are also assigned by a combination of SIIS and a partial deuteration strategy (Table 3, Supplementary Figure 5)^19,28^. In particular, the ^19^F resonance of F^2^, H-bonded to the K16 ammonium group, is differentially shifted by rotationally averaged HHH, HHD, HDD, and DDD congeners, leading to an unresolved peak in 10%, 50% and 95% D_2_O buffers. By contrast, F^3^, coordinated by two well-defined H-bonds from Y32 and the nucleophilic water, shows a resolved resonance at 50% D_2_O^19,28^.

**Table 3:** ^19^F NMR chemical shift (δ) comparison of GDP-BeF_3_^–^ GSA complexes and their respective linewidths in 10% D_2_O.

	Ras_WT_-GDP-BeF_3_^–^		Ras_Y32F_-GDP-BeF_3_^–^		Ras_pY64_-GDP-BeF_3_^–^	
	δ [ppm]	linewidth [ppm]^a^	δ [ppm]	linewidth [ppm]^a^	δ [ppm]	linewidth [ppm]^a^
F^1^	–176.3	0.45	–175.9	0.89	–176.9	0.57
F^2^	–152.5	0.50	–152.9	1.92	–152.0	0.82
F^3^	–160.5	0.51			–161.0	0.71

^a^Full width at half peak height.

The Ras_WT_-GDP-BeF_3_^–^ GSA complex exhibited a minor set of ^19^F signals comprising ~10% of the population, characterised by three resonances at –155.3 ppm, –162.6 ppm, and –183.0 ppm. Notably, all three resonances are shifted 3–7 ppm upfield relative to those of the major species (Fig. 4a), implying increased shielding for all three fluorines of BeF_3_^–^ in this minor species, a sign of the reduced extent of H-bonding to the BeF_3_^–^ moiety. To investigate whether this minor species originates from the “open” conformation that has been crystallised in the structure of Ras_Y32F_-GMPPNP (PDB: 3K9N), where the benzyl side chain adopts an “open” conformation, we introduced a Y32F mutation and generated the Ras_Y32F_-GDP-BeF_3_^–^ GSA complex, monitored using ^19^F NMR (Fig. 4b). Applying presaturation on the unbound fluoride resonance at –120 ppm was essential to distinguish the fluorine resonances associated with Ras from the free BeF_x_ resonances present in the solution around –167 ppm (Supplementary Figure 6). The ^19^F NMR spectrum of the Ras_Y32F_-GDP-BeF_3_^–^ GSA complex reveals a distinct overall profile: The trio of resonances, aligned with chemical shifts akin to the minor species in the Ras_WT_-GDP-BeF_3_^–^ GSA complex, emerged as predominant by integrations. In contrast, fluorine F^3^ from the second set of peaks, exhibiting chemical shifts more similar to the resonances of the “closed” conformation, has shifted downfield by 7.5 ppm and fused with F^2^ (Table 3). This is likely due to a water molecule assuming the role of an H-bond donor to F^3^, analogous to the phenolic -OH of Y32. This strongly underscores the pivotal role of Y32 -OH in neutralising the negative charge on the γ-phosphate during intrinsic GTP hydrolysis. It is noteworthy that, at equal component concentrations, the significantly lower signal-to-noise ratio in the Ras_Y32F_-GDP-BeF_3_^–^ GSA implies that the occupancy of BeF_3_^–^ is less than 100%, unlike the Ras_WT_-GDP-BeF_3_^–^complex, suggesting that Y32 -OH also contributes to the binding of both BeF_3_^–^ and GTP. The 10% population of Ras_WT_-GDP-BeF_3_^–^ GSA in “open” conformation explains why we could not crystallise this complex by co-crystallisation after many attempts and why its structure has been missing in the PDB.

### ^19^F NMR of metal fluoride complexes identify a structurally invisible effect of phosphorylation of Ras Y64

We next examined the impact of Y64 phosphorylation by investigating the Ras_pY64_-GDP-BeF_3_^–^ GSA complex via solution ^19^F NMR. The ^19^F NMR spectrum shows three major well-resolved resonances. Notably, they show small upfield shifts of 0.6 and 0.5 ppm for F^1^ and F^2^, respectively, compared to the Ras_WT_-GDP-BeF_3_^–^complex. Conversely, F^3^ experiences a 0.5 ppm downfield shift (Table 3, Fig. 4c). Although these chemical shift changes are marginal, they show only a small impact of Y64 phosphorylation on the catalytic site with no direct interaction existing between the BeF_3_^–^ moiety and the negatively charged phosphate on the phenolic -OH group of Y64. The average upfield shift of 0.2 ppm across the three resonances, compared to Ras_WT_-GDP-BeF_3_^–^, likely signifies a marginal increase in electron density on the three oxygen atoms within the γ-phosphate. This effect might induce a slightly increased repulsion during nucleophilic attack, thus rationalising the observed 3-fold reduction in intrinsic GTPase activity following phosphorylation. Moreover, the three resonances from the Ras_pY64_-GDP-BeF_3_^–^ complex have an average 0.70 ppm half-height line width, larger than the mean 0.48 ppm of the Ras_WT_-GDP-BeF_3_^–^ complex. This indicates a slightly slower exchange rate of the BeF_3_^–^ moiety with free fluoride and free BeF_x_ species in the Ras_pY64_-GDP-BeF_3_^–^ complex on the NMR time scale. Furthermore, a noteworthy but subtle observation emerges: the ratio between major and minor forms, calculated from the averaged integrations of F^1^ and F^2^, decreases by ~10%. This signals an increased ‘open’ conformation population in Ras_pY64_-GDP-BeF_3_^–^ following Y64 phosphorylation. Considering the pM affinity binding of GDP to Ras, the accelerated exchange rate of BeF_3_^–^ and increased population of open conformation further strengthens the assertion of a 2.6-fold increase in intrinsic nucleotide exchange rate upon phosphorylation (primarily on Y64), as also corroborated by NMR measurements^7^.

To investigate the impact of Y64 phosphorylation on GAP binding, we also attempted to form an MF_x_ TSA complex with Ras_pY64_ and RasGAP monitored by ^19^F NMR (Supplementary Figure 7). However, our attempts were unsuccessful, which stands in stark contrast to the easily formed RasGAP/Ras-GDP-“AlF_3_^0^” TSA complex (PDB: 1WQ1) observed by both crystallography^43^ and backed up by our ^19^F NMR test (Supplementary Figure 8). This confirms phosphorylation on Y64 could disrupt productive interactions with RasGAP, likely due to the steric hindrance because Y64 forms a H-bond with L902 of RasGAP in the RasGAP/Ras-GDP-AlF_3_^0^ complex structure.

### Effect of phosphorylation of tyrosine64 revealed by the structure of Ras_pY64_-GDP-BeF_3_^–^ GSA complex

To gain atomistic insight into how phosphorylation of Y64 could influence intrinsic GTP hydrolysis and nucleotide exchange rates with phosphorylated Ras, we initially assessed the stability of phosphorylated Ras. Our results indicated that mono-phosphorylated Ras remained stable in buffer for over a week, rendering it suitable for subsequent crystallisation trials (Supplementary Figure 9). We first crystallised the apo Ras_pY64_-GDP apo structure with commercial crystallisation screens. This structure was solved at 1.32 Å resolution (Table 1, PDB: 8BWG, Supplementary Data 2) but residues 60–64 as a key part of the Switch II loop do not have clear electron density. Thus, it cannot provide the accurate location and coordination of pY64. α2-Helix adopts a conformation that is highly similar to other GDP-bound product structures of Ras with an angle difference of <5° degree (Table 2).

**Fig. 5:**
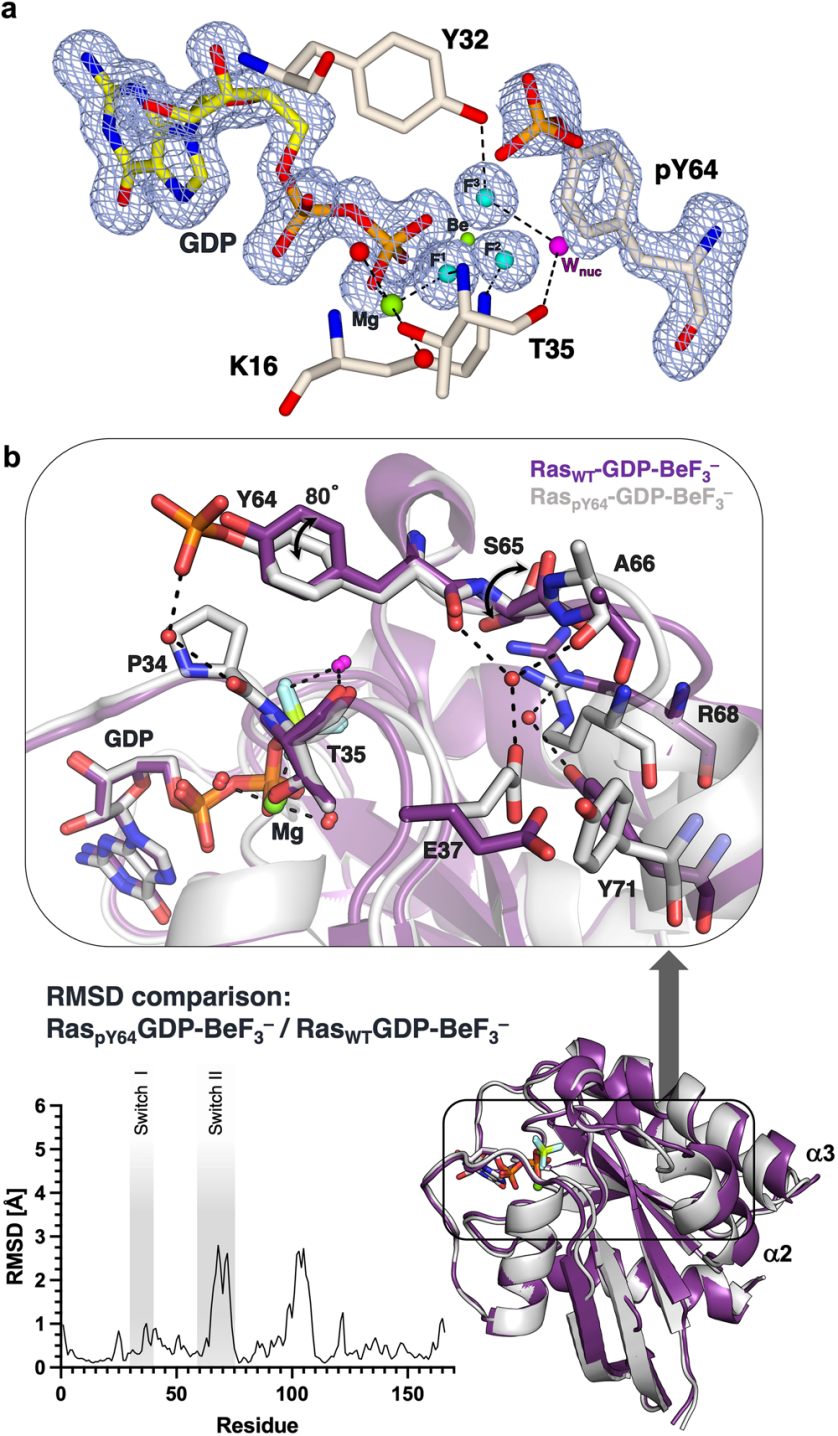
The structure of the Ras_pY64_-GDP-BeF_3_^–^ GSA complex. **a** Active site of Ras_pY64_-GDP-BeF_3_^–^. *F*_o_–*F*_c_ omit map for GDP, BeF_3_^–^ and pY64 are contoured at 3σ, 0.330 Å^–3^ (light blue mesh). Key neighbouring residues are shown in sticks. H-bonds coordinating to BeF_3_^–^ are shown as dashed lines. **b** Overlay of Ras_WT_-GDP-BeF_3_^–^ (purple) and Ras_pY64_-GDP-BeF_3_^–^ (grey). RMSD for the structure alignment is plotted for each residue.

We then obtained a BeF_3_^–^ GSA complex by soaking Ras_pY64_-GDP apo crystals with 50 mM BeCl_2_ and 0.8 M NH_4_F for 1 to 2 min before flash-freezing. The Ras_pY64_-GDP-BeF_3_^–^ GSA complex structure formed diffracts to 1.48 Å resolution (Table 1, PDB: 8CNN, Supplementary Data 3). While different from the Ras_pY64_-GDP apo structure, the Ras_pY64_-GDP-BeF_3_^–^ complex has well-defined electron densities for the flexible Switch II loop including the whole pY64, and the ordered Switch I loop in a “closed-up” conformation, where Y32 directly donates an H-bond to F^3^ (Fig. 5a, Supplementary Figure 10). Compared to the unphosphorylated Ras_WT_-GDP-BeF_3_^–^ GSA complex (backbone atom RMSD 0.766 Å, Supplementary Figure 11), our Ras_pY64_-GDP-BeF_3_^–^ GSA structure exhibits noticeable conformational changes (Fig. 5b). Originating from the phosphate in pY64, these extensive conformational shifts (spanning up to 28 Å) are a result of the alteration (80°) in the rotameric angle of the phenolic ring. This change is facilitated by an interaction with the backbone carbonyl group of Switch I P34, mediated through a water molecule. In turn, this changes the dihedral angle ψ (O–C–C_α_–N) of S65 from 39.3° to 173.5°, and the deviation of the α2-helix is further propagated throughout from the N-end of the α2-helix (residues 64–74) to the C-end of the α3-helix (residues 87–104) by a shift of by 2.5 Å (Fig. 5b). Indeed, T74 and M67 have shown noticeable chemical shift changes in ^1^H-^15^N HSQC in solution after phosphorylation^7^. The distinctive water-mediated interaction between the negatively charged phosphate on pY64 in Switch II and P34 in Switch I suggests its potential to obstruct the entry of a second water molecule into the active site. This observation offers another plausible rationale for the observed three-fold reduction in intrinsic hydrolysis rate^7^.

**Fig. 6:**
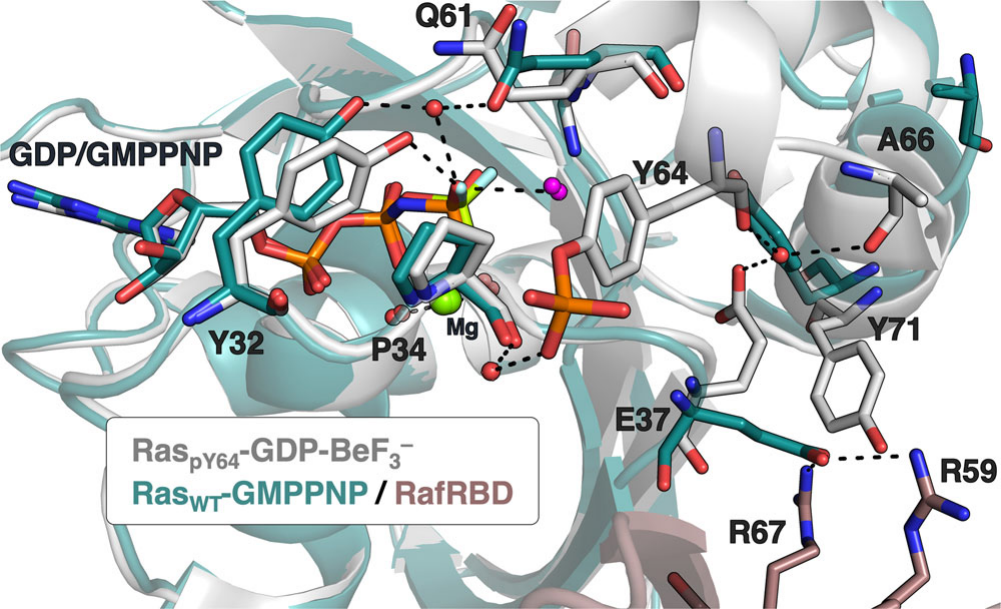
Insights of the impact of Y64 phosphorylation on Raf binding. Comparison of structures for Ras_pY64_-GDP-BeF_3_^–^ (PDB: 8CNN, grey) and Ras_WT_-GMPPNP/RafRBD (PDB: 4G0N, green/brown). Nucleophilic water is shown in magenta.

The diminished interaction between phosphorylated Ras and Raf-RBD has been explored by molecular dynamics, suggesting that the heightened flexibility of phosphorylated Y32 hinders Raf binding^7^. When Ras_pY64_-GDP-BeF_3_^–^ is aligned with Ras-GMPPNP-Raf complex structure (PDB: 4G0N), a noticeable alteration becomes evident. The backbone carbonyl group of pY64 reorients itself by forming a hydrogen bond with a water molecule, which simultaneously participates in H-bonding with the carbonyl group of A66 and the carboxylate sidechain of E37. The key interactions involving E37 of Ras and R59 and R67 of Raf are disrupted due to the substantial changes in the orientations of both E37 and Y71 following Y64 phosphorylation (Fig. 6). Our structure offers an additional explanation for the significant reduction in affinity between the downstream effector Raf and phosphorylated Ras, mediated by Src. This reduction is attributed to the extensive long-range conformational alterations resulting from the Y64 phosphorylation^7^.

## Conclusion

In this study, we have substantiated Y64 as the predominant site of phosphorylation by Src. This finding suggests that Ras likely exerts its biological function primarily in its mono-phosphorylated form. Making new use of pM GDP binding and readily available Be^2+^ and F^−^ salts, we successfully obtained a GDP-BeF_3_^−^ GSA complex for both unphosphorylated and Y64-phosphorylated Ras. The large conformational alterations induced by BeF_3_^–^ within the crystalline structure are of substantial significance. This demonstrates new efficacy and validity of a soaking approach in generating GDP-BeF_3_^–^ GSA complexes for small G proteins from their GDP-bound structures. It offers a convenient strategy for structural biological inquiries, without the need to carry out nucleotide exchange with non-hydrolysable GTP analogues. Our investigation reveals that the impact of Src-mediated Y64 phosphorylation on Ras extends beyond the Switch I and II regions. It encompasses broader steric and conformational effects, significant for the biological roles of this crucial PTM of Ras, especially for its interactions with effector proteins such as Raf. These two BeF_3_^–^ GSA complexes both capture a “closed” conformation where Y32 in Switch I directly interacts with Q61 in Switch II by H-bonding, different from other ground state conformations as depicted by caged-GTP, GMPPNP, GMPPCP, or GTPγS. Furthermore, the utility of ^19^F NMR is extended to elucidate the underlying molecular basis of conformational changes induced by PTMs, which might not be captured by high-resolution structures. It serves as a precedent for its application to the investigation of other PTMs, such as S-nitrosylation on C118^44^. Indeed, utilising ^19^F NMR on BeF_3_^–^ GSA complexes reveals that Switch I predominantly adopts a “closed” conformation ( ~ 90% occupancy) in solution. Our study underscores the utility of BeF_3_^–^ complexes to reveal distinct conformational nuances opaque with non-hydrolysable GTP analogues. Finally, BeF_3_^–^ GSA structures fill a crucial gap for molecular docking in Ras-targeted drug discovery and present valuable starting points for computational investigations, quantum mechanics/molecular mechanics (QM/MM) and molecular dynamics (MD), which can deliver disparate mechanistic and conformational insights.

## Methods

### Molecular cloning

pGEX-RasGAP334_(714–1047)_ encoding the human RasGAP domain (Uniprot: P20936) from residues 714 to 1047 and ptac-HRas_(1–166)_ encoding the human HRas (Uniprot: P01112) from residues 1 to 166 were kindly provided by the Wittinghofer lab^43^. ptac-HRas_(1–166)_-Y32F and ptac-HRas_(1–166)_-Y64F were generated by site-directed mutagenesis from ptac-HRas_(1–166)_ using PrimestarMax (Takara Bio Inc.) and sequenced to confirm the desired mutation. Plasmids pET28-cSrc_(251–533)_ encoding the gene of chicken Src kinase (Uniprot: P00523) catalytic domain (only three amino acids differ from its human homologue) and pCDFDUET-YoPH encoding a tyrosine phosphatase YoPH were a generous gift from Dr Feng Ni (Ningbo University, China)^45^.

### Gene expression and protein purification

HRas(1–166): *E. coli* BL21(DE3) cells were transformed with ptac-HRas_(1–166)_ plasmid, plated onto LB agar plates (100 μg/mL ampicillin) and grown overnight at 37 °C. A single colony was used to inoculate 30 mL of LB media (100 μg/mL ampicillin) and incubated at 37 °C overnight. This culture was used to inoculate LB medium (100 μg/mL ampicillin) at a 1:100 ratio. The culture was grown at 37 °C to an OD_600_ between 0.6 and 0.8 and gene expression was induced with 1.0 mM IPTG. The culture was shaken at 25 °C for 18 h and cells were harvested by centrifugation (7000 g, 4 °C, 20 min). The cell pellet was either stored at –80 °C or processed directly after centrifugation. The cell pellet was resuspended in buffer A (Tris-HCl 25 mM, pH = 7.6, MgCl_2_ 5 mM, DTT 1 mM) and supplemented with 1 mM PMSF. The cells were lysed by sonication (4 min sonication time, 2 s on and 8 s off) and cell debris was removed by centrifugation (32000 g, 4 °C, 40 min). The supernatant was filtered and loaded on a DEAE column, washed with 3 CV lysis buffer and eluted by applying a gradient of 0–100% elution buffer (Tris-HCl 25 mM, pH = 7.6, NaCl 200 mM, MgCl_2_ 5 mM, DTT 1 mM) over 8 CV. The eluted protein fractions were pooled and concentrated. Finally, the target protein was purified on a SEC75 26/60 column and stored at –80 °C. The same procedure was used for ptac-HRas_(1–166)_-Y32F and ptac-HRas_(1–166)_-Y64F.

RasGAP_334_(714–1047): *E. coli* BL21(DE3) cells were transformed with pGEX-2T-RasGAP_(714–1047)_ plated onto LB agar plates (100 μg/mL ampicillin) and grown overnight at 37 °C. A single colony was used to inoculate 30 mL of LB media (100 μg/mL ampicillin) and the culture was shaken at 37 °C overnight. This culture was used to inoculate LB media (ampicillin 100 μg/mL) at a 1:100 ratio. The culture was grown at 37 °C to an OD_600_ between 0.6 and 0.8 and expression was induced with 1.0 mM IPTG. The culture was incubated at 18° C for 18 h and cells were harvested by centrifugation (7000 *g*, 4 °C, 20 min). The cell pellet was resuspended in buffer A (Tris-HCl 50 mM, pH = 7.6, NaCl 150 mM, MgCl_2_ 5 mM, DTT 1 mM) and supplemented with 1 mM PMSF. Cells were lysed by sonication (4 min sonication time, 2 s on and 8 s off) and cell debris was removed by centrifugation (32000 *g*, 4 °C, 40 min). The supernatant was filtered and loaded on GST resin (GST-HP resin, Cytiva, United States). The resin was incubated on a tube roller at 4 °C for 60 min and washed with 5 CV buffer A. The target protein was eluted over 3 CV with buffer B (Tris-HCl 50 mM, pH = 7.6, NaCl 150 mM, MgCl_2_ 5 mM, DTT 1 mM, glutathione 10 mM) and buffer exchanged into buffer A. To cleave the GST-tag the protein solution was incubated at 4 °C with 25 NIH units of thrombin (T7326-1KU, Sigma Aldrich, United States). Progress of the cleavage reaction was controlled via SDS-PAGE and further thrombin was added as required. Upon completion of the cleavage reaction, the protein solution was incubated with GST resin at 4 °C. After 60 min the flow-through was collected and concentrated. Finally, the target protein was purified on a SEC75 26/60 column and stored at –80 °C.

Src(251–533): The catalytic domain of chicken Src was prepared following the protocol described in the literature^45^. *E. coli* BL21(DE3) cells were co-transformed with pET28-cSrc_(251–533)_ and pCDFDuet-YoPH and incubated on LB agar (50 μg/mL kanamycin and 50 μg/mL streptomycin) overnight at 37° C. A single colony was used to inoculate 30 mL of LB media (50 μg/mL kanamycin and 50 μg/mL streptomycin) and the culture was shaken at 180 rpm at 37 °C overnight. This culture was used to inoculate TB medium (50 μg/mL kanamycin and 50 μg/mL streptomycin) at a 1:100 ratio. The culture was grown at 37 °C to an OD_600_ between 1.0 and 1.2 and gene expression was induced with 0.2 mM IPTG. The culture was incubated at 18 °C for 16 h and cells were harvested by centrifugation (7000 g, 4 °C, 20 min). The cell pellet was either processed directly after centrifugation or stored at –80 °C. The cell pellet was resuspended in buffer A (Tris-HCl 50 mM, pH = 8.0, NaCl 500 mM, imidazole 25 mM, glycerol 5% (v/v)) and supplemented with 1 mM PMSF. Cells were lysed by sonication (4 min sonication time, 2 s on and 8 s off) and cell debris removed by centrifugation (32000 *g*, 4 °C, 40 min). The supernatant was filtered and loaded on a Ni^2+^-NTA column (Cytiva, 5 mL FF HisTrap). After washing with 5 CV buffer A, the target protein was eluted by applying a gradient of 0–50% buffer B (Tris-HCl 50 mM, pH = 8.0, NaCl 500 mM, imidazole 500 mM, glycerol 5% v/v) over 30 CV. The eluted protein fractions were pooled, concentrated, and dialysed at 4 °C overnight against 20 volumes of buffer C (Tris-HCl 20 mM, pH = 8.0, 100 mM, DTT 1 mM, glycerol 5% v/v). The crude kinase was then loaded onto a Q column (Cytiva, HiTrap FF 5 mL) and eluted by applying a gradient of 0–40% buffer D (Tris-HCl 20 mM, pH = 8.0, NaCl 1.0 M, DTT 1 mM, glycerol 5% v/v). Fractions containing the kinase were pooled, further purified on a SEC75 16/60 column and stored at –80 °C.

### Ras phosphorylation assay by Src

500 µM Ras-GDP was incubated for 2 h at 25 °C in assay buffer (Tris-HCl 25 mM pH = 7.6, NaCl 150 mM, MgCl_2_ 5 mM, DTT 1 mM, ATP 4 mM, cSrc 20 µM). To remove excess ADP/ATP and quantify each Ras species, monophosphorylated Ras as the major species shown by the chromatogram was separated from unphosphorylated and double-phosphorylated species by ion-exchange chromatography on a 16 × 100 mm Q FF 16/10 column. The chromatogram shows a major monophosphorylated peak, and two double-phosphorylated peaks as identified by MS. (Supplementary Figure 2, Supplementary Figure 12).

### Mass spectrometry (MS)

Ras_WT_, Ras variants and all the phosphorylated Ras species were subjected to MS for confirmation (Supplementary Figure 12). Liquid chromatography-mass spectrometry (LC-MS) was performed on a WATERS Synapt G2-Si quadrupole time-of-flight mass spectrometer coupled to a WATERS Acquity H-Class ultraperformance liquid chromatography (UPLC) system. The column was a WATERS Acquity UPLC protein BEH C4 (300 Å, 1.7 μm × 2.1 mm × 100 mm) operated in reverse phase and held at 60 °C. The gradient employed was 95% A to 35% A over 50 min, where A is water with 0.1% HCO_2_H and B is acetonitrile with 0.1% HCO_2_H. Spectra were collected in positive ionisation mode and analysed using WATERS MassLynx software version 4.1. Deconvolution of protein-charged states was obtained using the maximum entropy 1 processing software.

### Nuclear Magnetic Resonance (NMR) spectroscopy

NMR spectra in this work have been recorded on a 500 MHz Bruker five-channel liquid-state spectrometer with a high sensitivity QXI cryoprobe. Chemical shifts (δ) are given in parts per million (ppm). All spectra were recorded at 20 °C. For the presaturation of the free fluoride signal, elective ^19^F irradiation was achieved with a continuous wave (power level of 42 dB) applied over the 1 s recycle delay at the frequency of free fluoride peak (–119.5 ppm). For samples with 90% D_2_O, this frequency was adjusted to –121.5 ppm. Unless stated otherwise, all protein ^19^F NMR spectra were calibrated to an internal fluorobenzene standard at –113.79 ppm^46^.

### Stability of phosphorylation on Ras tyrosine

The stability of phospho-Ras was determined by incubating 100 μM monophosphorylated Ras-GDP in crystallisation buffer (HEPES-Na 20 mM, pH = 8.0, MgCl_2_ 10 mM, NaF 20 mM). At regular intervals, 30 μL aliquots were taken and mixed with SDS-PAGE loading buffer, heated to 95 °C for 3 min and stored at –80 °C until all time points could be analysed by SDS-PAGE (Supplementary Figure 9).

### Protein x-ray crystallography

Protein crystallisation conditions were set up either using a Douglas Instruments ORYX4 or a SPTlabtech Mosquito Crystal system in either a hanging drop or sitting drop configuration. Microseeding was performed based on literature conditions^47^.

Ras_pY64_-GDP: Condition screening using a commercial crystal screen (Hampton Research, HR2-130) was initially for searching for a condition for the “Ras_pY64_-GDP-RasGAP_334_” complex but yielded a hit for Ras_pY64_-GDP under sitting drop conditions (drop size 600 nL) with a 1:1 ratio of protein solution (Ras_pY64_-GDP 400 μM, RasGAP_334_ 400 μM, Na-HEPES 20 mM pH = 8.0, MgCl_2_ 5 mM, NaF 20 mM) and precipitant (Na-citrate 100 mM pH = 5.6, Li_2_SO_4_ 1.0 M, CaCl_2_ 200 mM). After three rounds of microseeding well-formed single crystals were obtained using 2.0 μL sitting drops and a 1:1 ratio of protein stock (Ras_pY64_-GDP 400 μM, RasGAP_334_ 400 μM, Na-HEPES 20 mM pH = 8.0, 5 mM MgCl_2_, NaF 20 mM) and precipitant (Na-citrate 100 mM pH = 5.6, Li_2_SO_4_ 800 mM, CaCl_2_ 200 mM). These were harvested using cryoprotectant (80% precipitant, 20% glycerol v/v) and used for data collection.

### Metal fluoride soaking

Ras_pY64_-GDP-BeF_3_^–^ GSA complex: To obtain the Ras_pY64_-GDP apo crystals, protein stock (Ras _pY64_ 400 µM, Na-HEPES 20 mM pH = 8.0, MgCl_2_ 5 mM, NaF 20 mM) was mixed with precipitant (100 mM NaOAc, pH = 4.5, 200 mM Li_2_SO_4_, 50% v/v PEG400) in a 1:1 ratio with a total drop size of 600 nL. Protein crystals were then soaked in the precipitant solution supplemented with 50 mM BeCl_2_ and 800 mM NH_4_F.

Ras-GDP-BeF_3_^–^ GSA complex: Protein stock (Ras-GDP 400 µM, Na-HEPES 20 mM pH = 8.0, MgCl_2_ 5 mM, NaF 20 mM) was mixed with precipitant (30% v/v MPD, 100 mM imidazole, pH = 7.0) in a 1:1 ratio with a total drop size of 600 nL. Protein crystals were then soaked in the precipitant solution supplemented with 50 mM BeCl_2_ and 800 mM NH_4_F.

The soaked crystals were subsequently flash-frozen using cryoprotectant (80% precipitant, 20% glycerol v/v) before data collection.

### Data collection, structure solution and refinement

The datasets described in this report were collected at the Diamond Light Source, Didcot, Oxfordshire, U.K. on beamline I03. Data were integrated using XDS^48^ and scaled/merged using AIMLESS^49^ included in the CCP4 software suite xia2^50^. Data collection and refinement statistics are provided in Table 1. The structures were solved by molecular replacement using MOLREP^51^ with one monomer of Ras in PDB: 1WQ1 as the model and refined with Refmac^52^.

Backbone RMSD for each pair of Ras structures were calculated by aligning the structures in PyMOL and then visualised using a Putty model. (https://github.com/tongalumina/rmsdca/blob/5a5e55ef97170bfbb5c6a66fdb214b76d3887519/rmsdCA.py).

### Reporting summary

Further information on research design is available in the Nature Portfolio Reporting Summary linked to this article.

### Supplementary information

Supplementary Information

Description of Additional Supplementary Files

supplementary data 1

supplementary data 2

supplementary data 3

Reporting Summary

## Data Availability

Ras_WT_-GDP-BeF_3_^–^, Ras_pY64_-GDP apo, and Ras_pY64_-GDP-BeF_3_^–^ structures have been deposited in the Protein Data Bank (PDB) with the accession codes **8CNJ** (Supplementary Data 1), **8BWG** (Supplementary Data 2), and **8CNN** (Supplementary Data 3), respectively.
