# Supplementary material for: Far-reaching effects of tyrosine64 phosphorylation on Ras revealed with BeF3– complexes
Source: Commun Chem. 2024 Jan 31;7:19. doi: 10.1038/s42004-024-01105-6 (PMC10830474; doi:10.1038/s42004-024-01105-6)
Supplement: Supplementary file 1 — Supplementary Information [file 42004_2024_1105_MOESM1_ESM.pdf]

Supporting Information:

Far-reaching Effects of Tyrosine64 Phosphorylation on Ras Revealed with BeF_3^- Complexes

Patrick Baumann[1,2] and Yi Jin*[1,2]

[1] School of Chemistry, Cardiff University, Park Place, Cardiff, CF10 3AT, United Kingdom

[2] Present address: Manchester Institute of Biotechnology, University of Manchester, 131 Princess Street, Manchester, M1 7DN, United Kingdom

* E-mail: yi.jin@manchester.ac.uk

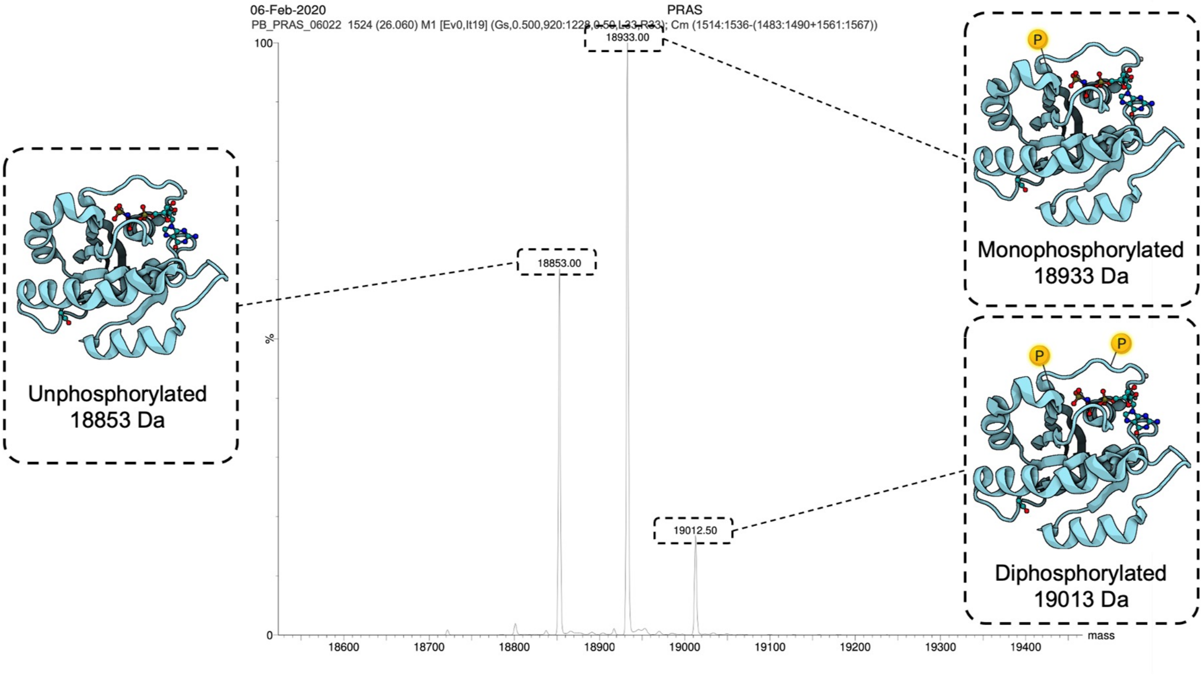

Supplementary Figure 1. Mass spectroscopy (MS) analysis of Src-mediated phosphorylation of Ras**.**

HRas (1.0 mM) was incubated with cSrc (20 μM) for 15 h at 4 ˚C in phosphorylation buffer (Tris-HCl 25 mM, pH = 7.6, NaCl 200 mM, 2-ME 2 mM, $MgCl_2$ 5 mM, ATP 5 mM). The deconvoluted spectrum detected unphosphorylated, mono- and double-phosphorylated species after the Src phosphorylation assay.

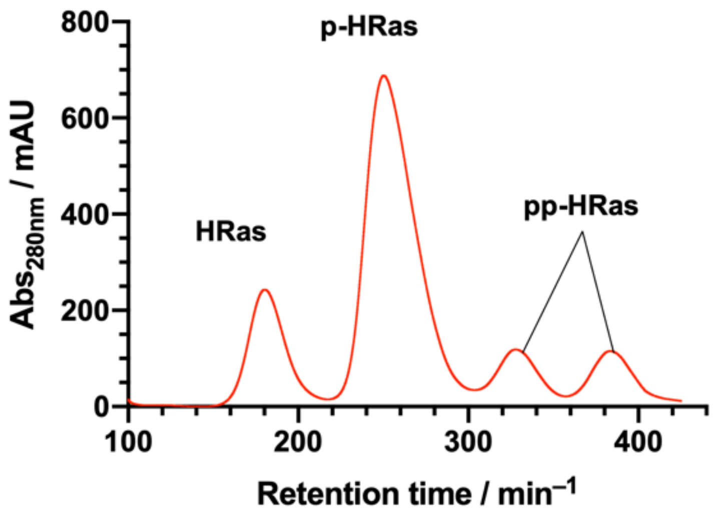

Supplementary Figure 2. Separation of phosphorylated Ras species by anion exchange chromatography.

Separation of Src-phosphorylated HRas into un-, mono- and double-phosphorylated species using anion exchange chromatography. After overnight incubation of 1.0 mM HRas with Src (1:250 Src:HRas molar ratio) in phosphorylation buffer (Tris-HCl 25 mM, pH = 7.6, NaCl 200 mM, 2-ME 2 mM, $MgCl_2$ 5 mM, ATP 4 mM) at 4 ˚C, the unmodified, singly phosphorylated and doubly phosphorylated forms were separated using anion exchange chromatography. A 16x100 mm Q FF 16/10 column was run with 20 mM HEPES pH = 7.0, 5 mM $MgCl_2$, and 1 mM DTT (Buffer A), and 20 mM HEPES pH = 7.0, 5 mM $MgCl_2$, 1mM DTT, and 1M NaCl (Buffer B) using a gradient of 0 to 40% B over 80 column volumes.

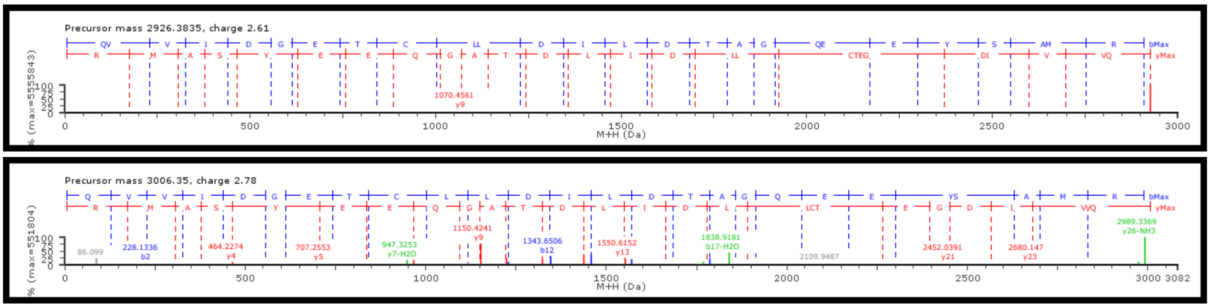

Supplementary Figure 3. Trypsin digest of monophosphorylated Ras.

Data was acquired using an Agilent 6520 Ion Mobility LC/Q-TOF system in positive mode (reference mass 922.00979800) equipped with a LC Agilent 1290 Infinity system and Waters ACQUITY UPLC glycoprotein BEH Amide (300 Å, 1.7 µm) column. Column flow was set at 0.2 mL/min. MS/MS spectrum of native and phosphorylated-peptides (light chain peptide 1-18). Top (native peptide): 2926.38. Bottom (Y64-phosphorylated fragment): 3006.35. Fragment sequence: RMAS**Y***EEQGATDLIDLLCTRGDIVVQ.

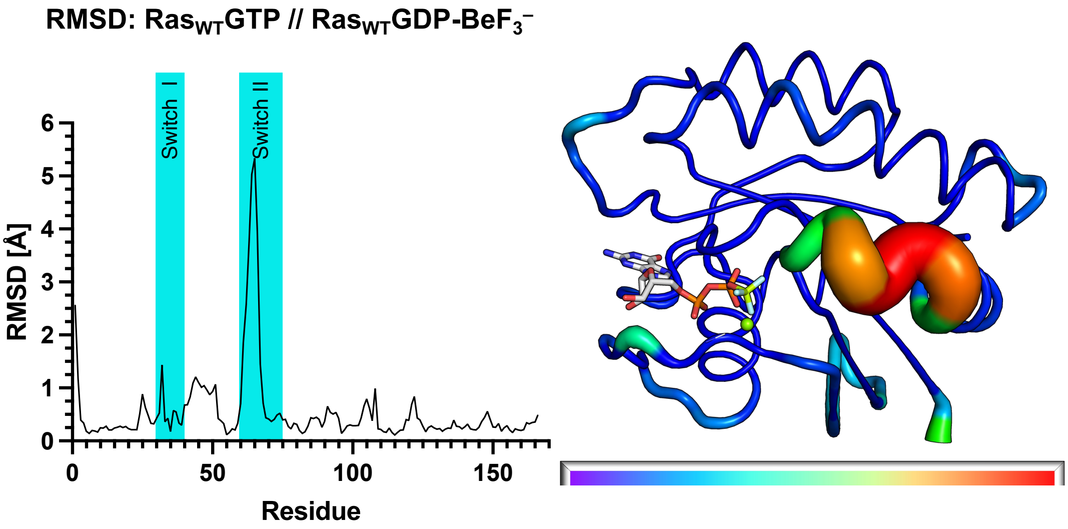

Supplementary Figure 4. RMSD comparison of structures of Ras$_{WT}$-GTP (PDB: 1QRA) with Ras$_{WT}$-GDP-BeF_3^- (PDB: 8CNJ) on the backbone.

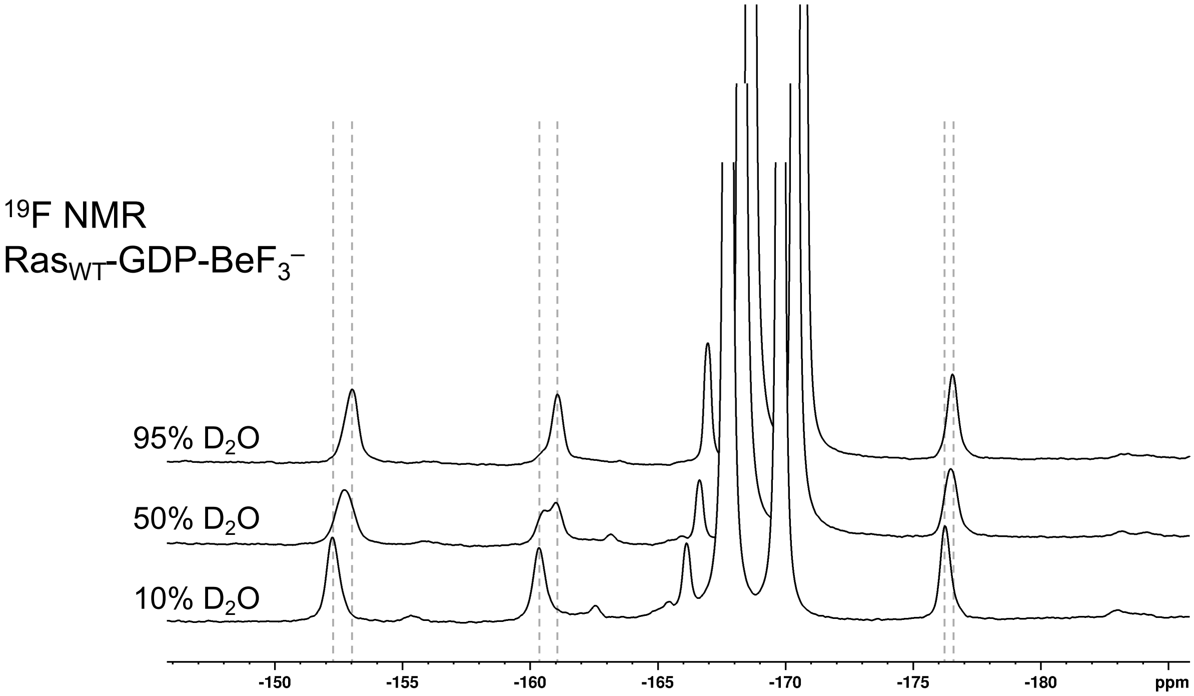

Supplementary Figure 5. Solvent-induced isotope shifts (SIIS) analysis of Ras$_{WT}$-GDP-BeF_3^- using ^{19}F-NMR.

^{19}F NMR, 500 MHz, 2048 scans: Ras 1.1 mM, Tris-HCl 25 mM, pH/pD = 7.5, NaCl 150 mM, $MgCl_2$ 5 mM, NH_4F 30 mM, DTT 1 mM, $BeCl_3$ 3 mM. NMR samples contain 10–95% D_2O content.

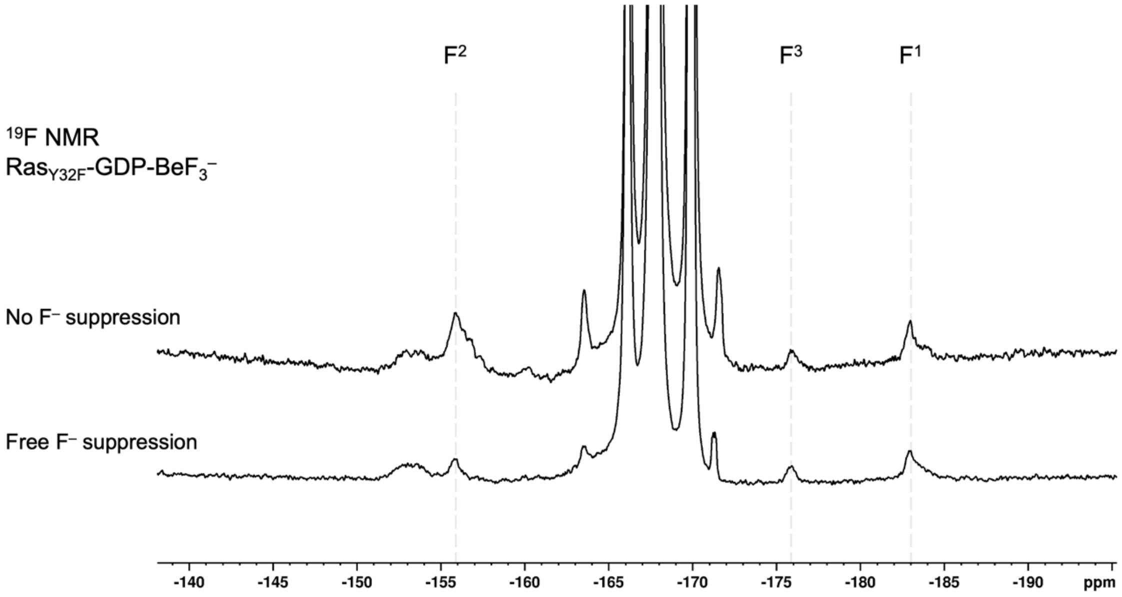

Supplementary Figure 6. ^{19}F NMR Ras_{Y32F}-GDP-BeF_3^- spectrum. Free fluoride presaturated on –120 ppm to suppress the protein-unbound BeF_x species (bottom) compared to unpresaturated spectrum (top).

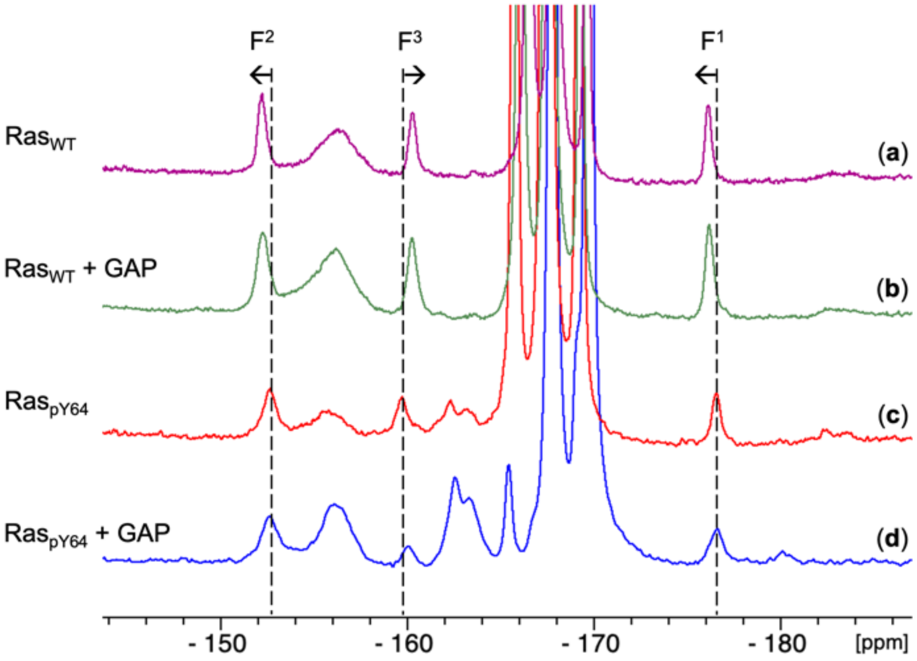

Supplementary Figure 7. ^{19}F NMR spectra for Ras-BeF_3^- complexes. ^{19}F NMR spectra with 1024 scans were recorded for **(a)** Ras_{WT}-GDP-BeF_3^- GSA complex; **(b)** NMR sample of (a) supplemented with 1.1 eq of $RasGAP_{334}$; **(c)** $HRas_{pY64}$-GDP-BeF_3^- GSA complex; **(d)** NMR sample of (c) supplemented with 1.1 eq of $RasGAP_{334}$. All samples consisted of 0.8–1.0 mM Ras_{WT}, Ras_{Y32F} or $HRas_{pY64}$, in the buffer of 25 mM Tris-HCl pH = 7.0 and 10% D_2O with 3 mM $BeCl_2$, 30 mM NH_4F, and 5 mM $MgCl_2$.

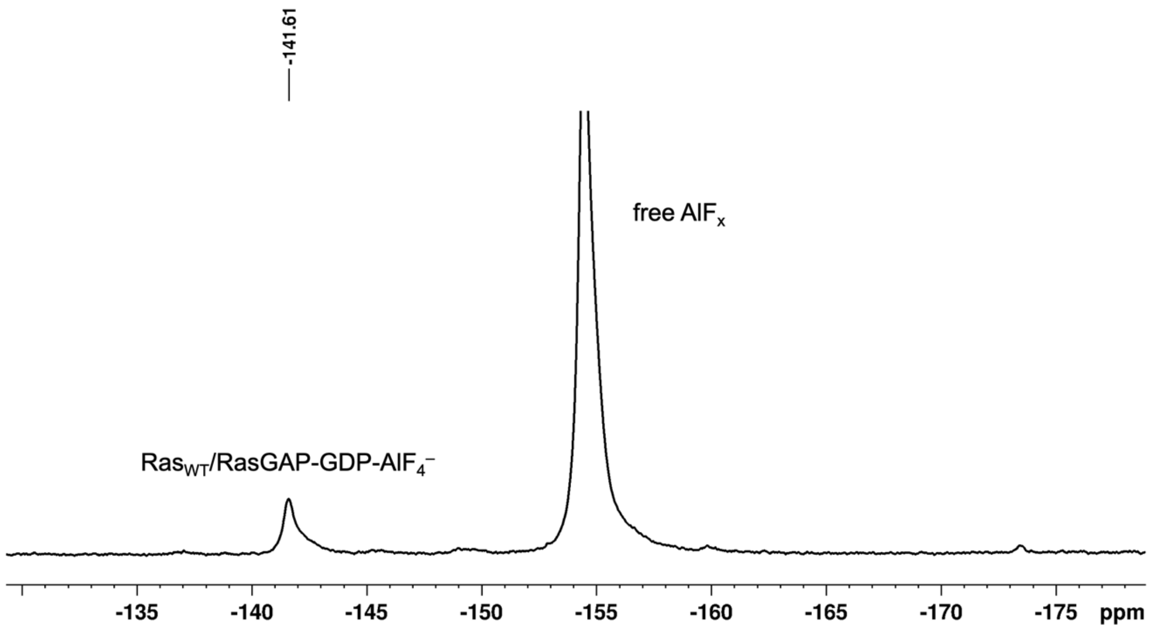

Supplementary Figure 8. ^{19}F NMR spectrum of RasGAP$_{334}$/HRas$_{WT}$-GDP-AlF$_4^-$ TSA complex.
^{19}F NMR, 500 MHz, 1024 scans: Ras 1.0 mM, Tris-HCl 25 mM, pH = 7.5, NaCl 200 mM, $MgCl_2$ 5 mM, NH_4F 30 mM, DTT 1 mM, $AlCl_3$ 3 mM, D_2O 10%.

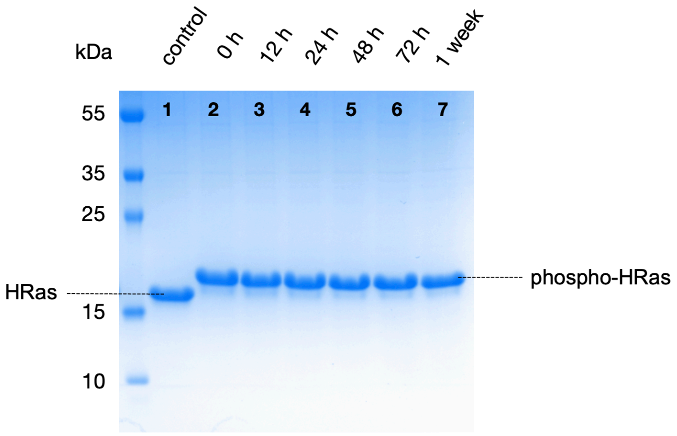

Supplementary Figure 9. Stability of monophosphorylated HRas.
The stability of monophosphorylated Ras was determined by incubating 100 µM monophosphorylated HRas in crystallisation buffer (HEPES-Na 20 mM pH = 8.0, $MgCl_2$ 10 mM, NaF 20 mM) at 4 ˚C. In regular intervals aliquots were taken, mixed with SDS-PAGE loading buffer, heated to 100 ˚C for 3 min and stored at –80 ˚C until all timepoints could be analysed by SDS-PAGE.

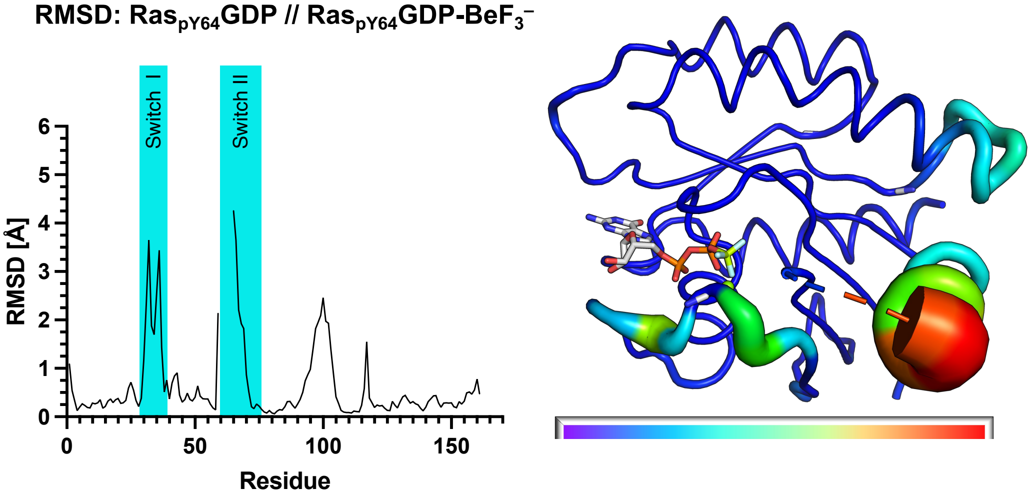

Supplementary Figure 10: RMSD comparison of the structures of Ras$_{pY64}$-GDP (PDB: 8BWG) with Ras$_{pY64}$-GDP-BeF$_3^-$ (PDB: 8CNN) on the backbone.

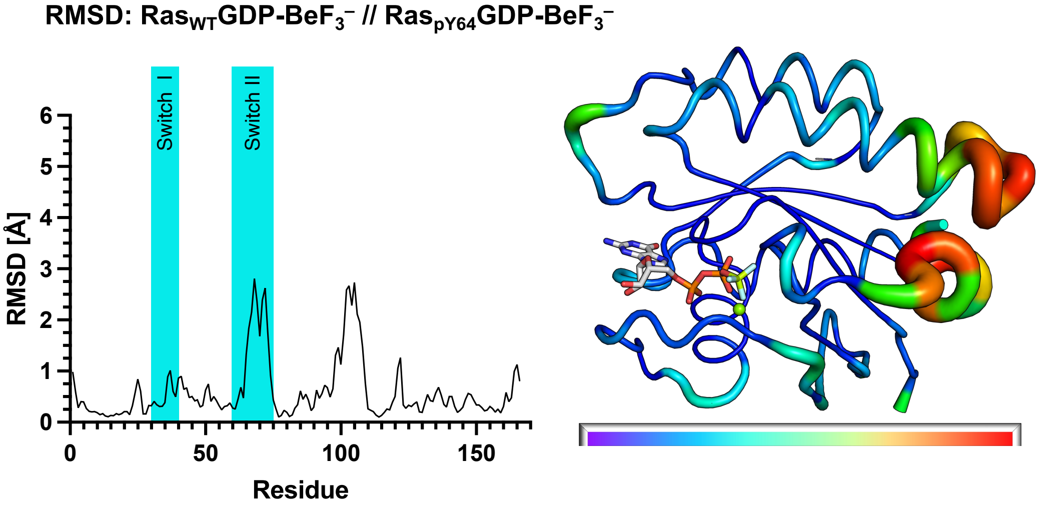

Supplementary Figure 11: RMSD comparison of structures of Ras$_{WT}$-GDP-BeF$_3^-$ (PDB: 8CNJ) was aligned on Ras$_{pY64}$-GDP-BeF$_3^-$ (PDB: 8CNN).

Mass spectrum for $HRas_{WT}$

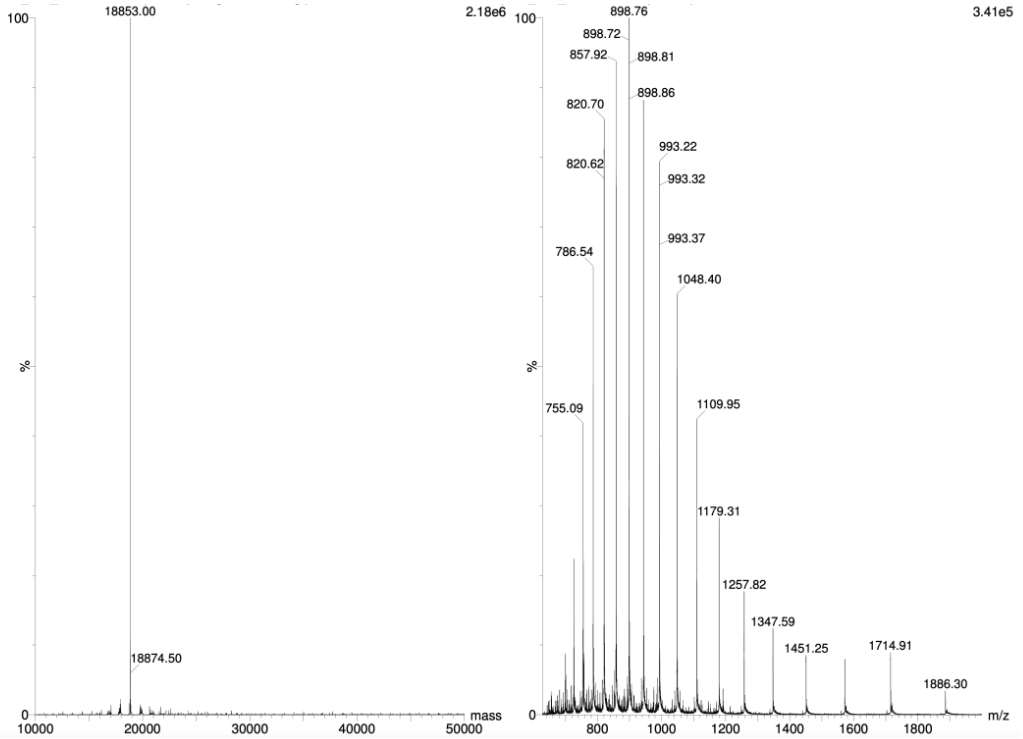

Mass spectrum for monophosphorylated Ras_{WT}.

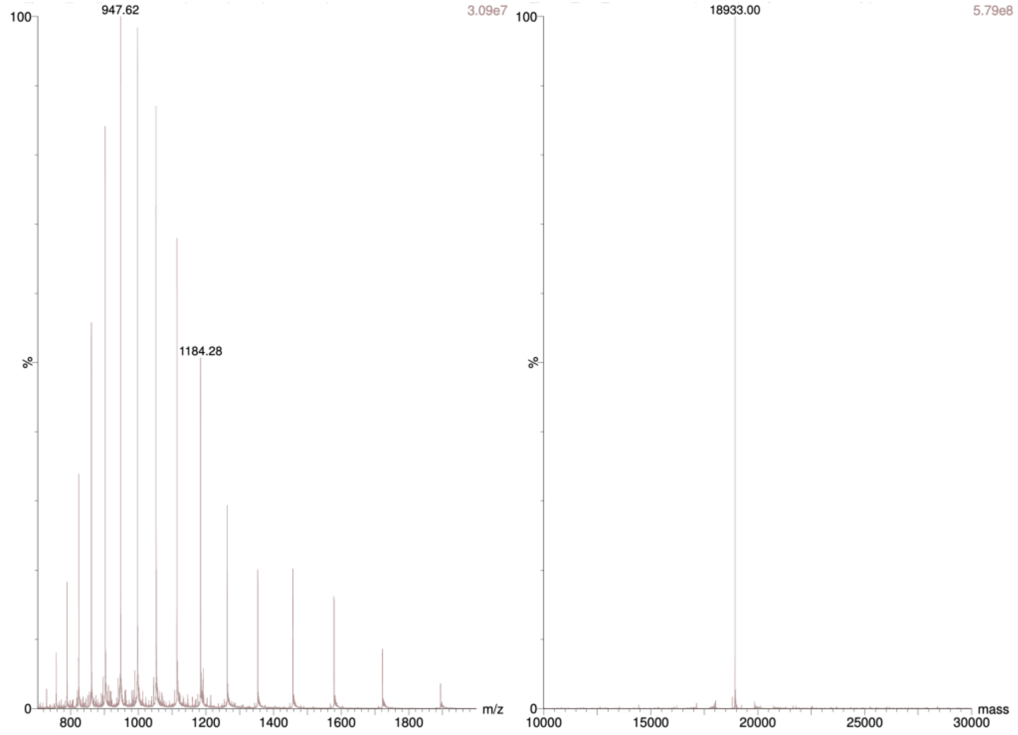

Mass spectrum for double-phosphorylated Ras_{WT}

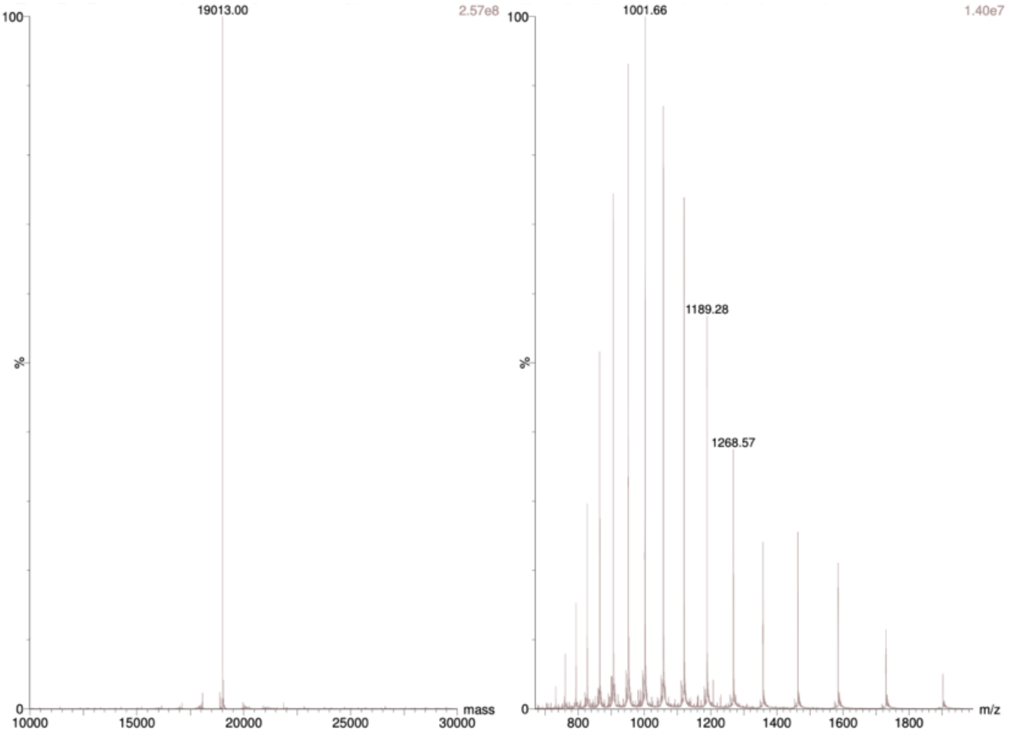

Mass spectrum for Ras_{Y32F}

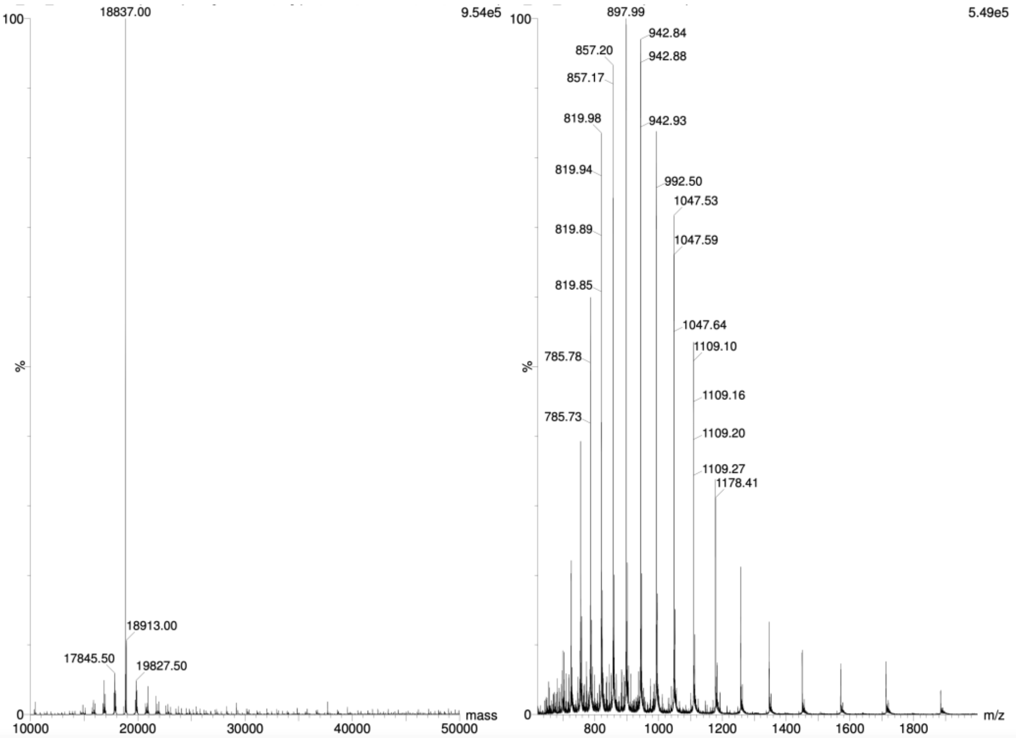

Mass spectrum for Ras_{Y64F}

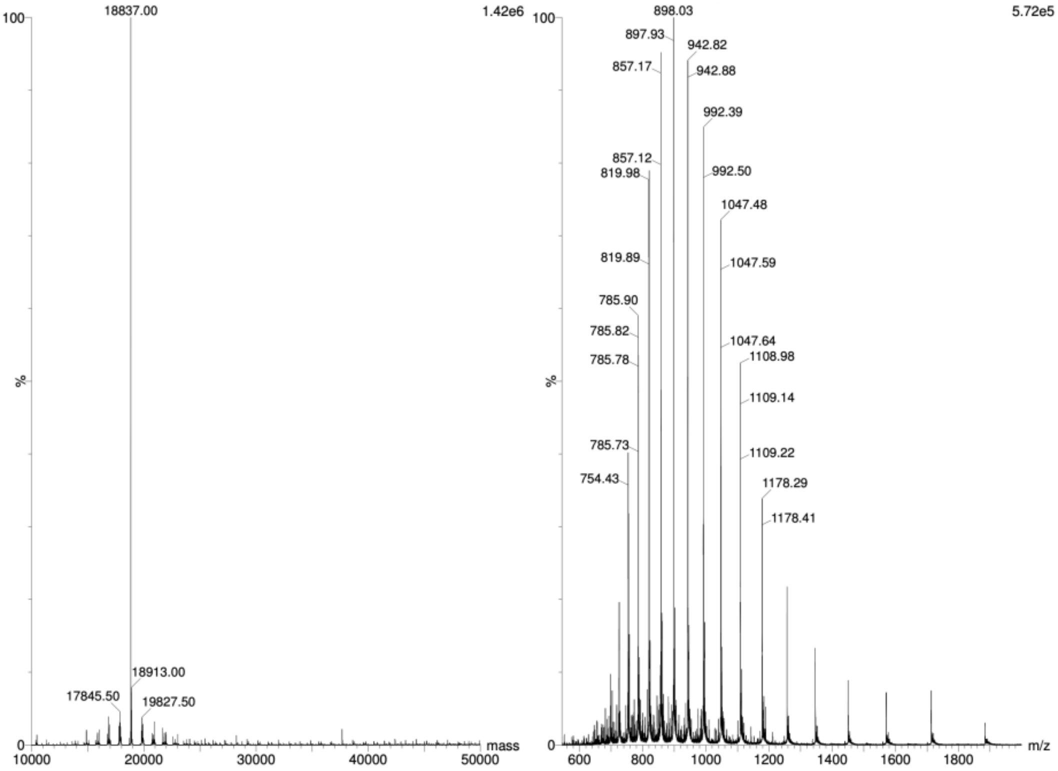

Supplementary Figure 12: Protein mass spectrometry for Ras_{WT}, Ras_{Y32F}, Ras_{Y64F}, mono-phosphorylated Ras_{WT} and double-phosphorylated Ras_{WT}.
