# Supplementary material for: Far-reaching effects of tyrosine64 phosphorylation on Ras revealed with BeF3– complexes
Source: Commun Chem. 2024 Jan 31;7:19. doi: 10.1038/s42004-024-01105-6 (PMC10830474; doi:10.1038/s42004-024-01105-6)
Supplement: Supplementary file 2 — Description of Additional Supplementary Files [file 42004_2024_1105_MOESM2_ESM.pdf]

Description of Additional Supplementary Files

File name: Supplementary Data 1

Description: The PDB file for the macromolecule structure of Ras_{WT}-GDP-BeF_3^- GSA complex, PDB:**8CNJ**

File name: Supplementary Data 2

Description: The PDB file for the macromolecule structure of Ras_{pY64}-GDP apo, PDB:**8BWG**

File name: Supplementary Data 3

Description: The PDB file for the macromolecule structure of Ras_{pY64}-GDP-BeF_3^-, PDB: **8CNN**
